# Control of Helical Navigation by Three-Dimensional Flagellar Beating

**DOI:** 10.1103/PhysRevLett.126.088003

**Published:** 2021-02-26

**Authors:** Dario Cortese, Kirsty Y. Wan

**Affiliations:** Living Systems Institute and College of Engineering, Mathematics and Physical Sciences, University of Exeter, Exeter EX4 4QD, United Kingdom

## Abstract

Helical swimming is a ubiquitous strategy for motile cells to generate self-gradients for environmental sensing. The model biflagellate *Chlamydomonas reinhardtii* rotates at a constant 1–2 Hz as it swims, but the mechanism is unclear. Here, we show unequivocally that the rolling motion derives from a persistent, nonplanar flagellar beat pattern. This is revealed by high-speed imaging and micromanipulation of live cells. We construct a fully 3D model to relate flagellar beating directly to the free-swimming trajectories. For realistic geometries, the model reproduces both the sense and magnitude of the axial rotation of live cells. We show that helical swimming requires further symmetry breaking between the two flagella. These functional differences underlie all tactic responses, particularly phototaxis. We propose a control strategy by which cells steer toward or away from light by modulating the sign of biflagellar dominance.

Organisms perceive their world as three dimensional. Biological swimmers adopt chiral or helical trajectories to navigate through bulk fluid. Helical navigation is a ubiquitous locomotion strategy found across diverse taxa and multiple length scales, from the planktonic larvae of marine invertebrates [1] to small protists [2] and spermatozoa [3]. Bacteria bias the rotation of chiral flagella in response to chemical gradients [4]. In contrast, many unicellular eukaryotes *steer* toward or away from external stimuli (light, chemicals, gravity) via helical klinotaxis and a corkscrewing motion around their body axis. Such organisms routinely integrate sensory information obtained by subcellular sensors (receptors, eyespots), which periodically scan the environment, with motor actuators (cilia, flagella), to adjust their swimming trajectories according to the stimulus. These self-actions can enhance signal perception at the microscale [5], with important consequences for evolution and eukaryogenesis [6]. Helical movement could help compensate for asymmetries in body shape [7] and/or filament actuation [8], but it is unclear whether such asymmetries evolved as an adaptation to helical swimming or to facilitate it in the first place. Self-propelled cholesteric liquid crystal droplets also exhibit curling and helical motions, depending on an interplay between surface flows and nematic order [9]. The complex motility strategies of real cells can facilitate the development of next-generation artificial swimmers and controllable devices [10].

For algal flagellates, the control of flagellar beating is key to effective spatial navigation and reorientation to photostimuli [11–13]. Spermatozoa steer via changes to their 3D beat pattern [14], while ciliates tilt the orientation of ciliary fields to turn [15]. *Chlamydomonas* swims with two near-identical flagella using a characteristic breast-stroke, rotating slowly (1–2 Hz) about its axis as it swims along left-handed helices [16,17]. Each (~10 *μ*m) cell has a unique photosensor (eyespot), fixed in position on the rigid cell body [18]. The eyespot scans the environment and modulates phototactic turning by periodic shading [19,20]. Yet to date, there has been no compelling explanation for this characteristic helical motion. Here, we combine theory and experiment to reveal how *Chlamydomonas*, despite a very symmetric body plan, manipulates nonplanar biflagellar beating to achieve helical swimming and stimulus-dependent steering responses in 3D. In contrast, other microswimmers rely on obvious body asymmetries to steer [21], such as two distinct flagella types in some dinoflagellates [22].

## Model formulation

**Fig. 1:**
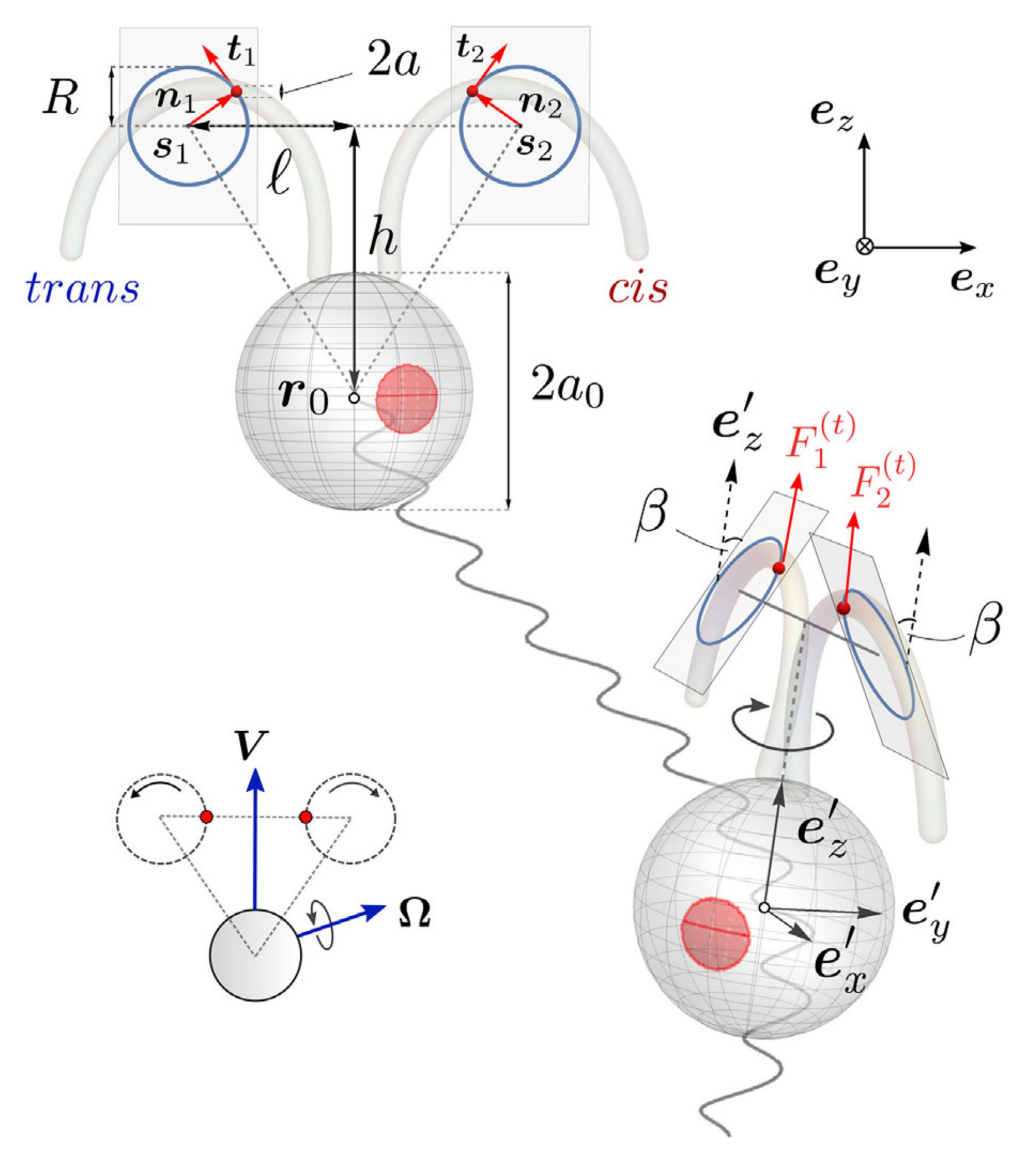
A fully 3D model of a freely swimming *Chlamydomonas* cell, in front and side views. Flagellar beating is modeled by small beads constrained to rotate along circular orbits embedded within a pair of tilted beat planes, for a realistic scaffold geometry (inset). $\left\{e_{x}^{\prime },e_{y}^{\prime },e_{z}^{\prime }\right\}$ and $\left\{e_{x},e_{y},e_{z}\right\}$ are the body and laboratory reference frames.

Our fully 3D model of the swimmer comprises three beads interacting hydrodynamically in an incompressible Newtonian fluid at zero Reynolds number. The cell body is a sphere of radius *a*_0_, located at ***r***_0_. Two smaller beads (radius *a* ≪ *a*_0_) are localized at the centers of drag of the flagella ***r***_*i*_, *i =*1, 2. Flagella beads are driven by variable tangential forces $F_{i}^{\left(t\right)},i=1,2$ and constrained along circular orbits of radius *R* by normal components $F_{i}^{\left(n\right)}$ , mimicking breaststroke swimming. All three beads are constrained on a rigid triangular scaffold at rest with the body frame of reference (Fig. 1), with dimensions *l* and *h*, and *a* ≪ *a*_0_ ≪ *h*, 𝓁. This is motivated by experiments that show that flow fields induced by freely swimming *Chlamydomonas* cells are well described by just three Stokeslets [23]. In-plane versions of these models recapitulated the stochastic (run-and-tumble) character of biflagellar coordination [24–27]. These minimal representations of ciliary beating as beads moving along a prescribed orbits are powerful tools for studying hydrodynamic synchronization [28–30].

Unlike in previous studies, we allow the flagellar beads to rotate out of the plane. This introduces a nonzero tilt angle *β* between the flagellar orbital planes π_(±*β*)_ and the frontal $e_{x}^{\prime }-e_{z}^{\prime }$ plane, which we hypothe suffices to produce both axial rotation and helical swimming [Fig. 1(b)]. The flagella orbits are centered at $s_{i}=r_{0}+{\left(-1\right)}^{i}\ell e_{x}^{\prime }+he_{z}^{\prime },\;i=1,2$ Flagellar beads, located at ***r***_*i*_ = *s*_*i*_ + *R****n***_*i*_, with $n_{i}={\left(-1\right)}^{i+1}\cos\varphi_{i}e_{x}^{\prime }+\;{\left(-1\right)}^{i+1}\sin\beta \sin\varphi_{i}e_{y}^{\prime }+\cos\beta \sin\varphi_{i}e_{z}^{\prime },$ rotate with phases *φ*_*i*_. For breaststrokes, we require $0\leq \beta <\pi /2,{\dot{\varphi }}_{i}\geq 0$. The body axes $e_{i}^{\prime }$ transform to the lab frame ***e***_*i*_ via Euler angles ***θ*** =(*θ*_1_, *θ*_2_, *θ*_3_) (pitch, yaw, and roll). The swimmer kinematics are fully described by ***X*** =(*x*_0_, *y*_0_, *z*_0_, *θ*_1_, *θ*_2_, *θ*_3_, *φ*_1_, *φ*_2_) and the parameters *h, l, β*, *R, a*_0_, *a*. We impose force- and torque-free conditions: ***F***_0_ + ***F***_1_ + ***F***_2_ = **0** and ***T***_0_ + ***T***_1_ + ***T***_2_ = **0**, where $T_{i}=r_{i}\times F_{i}+{\bar{T}}_{i}$ and ${\bar{T}}_{i}$ is the intrinsic torque due to the *i*th sphere’s rotation around an internal axis. Since *a* ≪ *a*_0_, we assume that ${\bar{T}}_{1,2}\ll {\bar{T}}_{0}$. The swimmer moves with velocity $V={\dot{r}}_{0}$ and angular velocity **Ω**. Using the Oseen approximation for hydrodynamic interactions between beads [31], we have

(1) $$V=\left[G\left(r_{01}\right)-\gamma_{0}^{-1}\right]\cdot F_{1}+\left[G\left(r_{02}\right)-\gamma_{0}^{-1}\right]\cdot F_{2},$$

(2) $${\dot{r}}_{1}=\gamma_{1}^{-1}F_{1}+\left[G\left(r_{12}\right)-G\left(r_{10}\right)\right]\cdot F_{2}-G\left(r_{10}\right)\cdot F_{1},$$

(3) $${\dot{r}}_{2}=\gamma_{2}^{-1}F_{2}+\left[G\left(r_{21}\right)-G\left(r_{20}\right)\right]\cdot F_{1}+G\left(r_{20}\right)\cdot F_{2},$$

(4) $$\Omega =\gamma_{0r}^{-1}{\bar{T}}_{0}=-\gamma_{0r}^{-1}\left(T_{1}+T_{2}\right),$$

where $r_{ij}=r_{i}-r_{j},\;\gamma_{i}=6\pi \eta a_{i},\;\gamma_{0r}=8\pi \eta a_{0}^{3},\;G(r)=\;(I+\hat{r}⊗\hat{r})/(8\pi \eta )$, and the dot denotes a time derivative. These reduce to a set of ten equations for unknowns $\left(V,\Omega ,{\dot{\varphi }}_{1,2},F_{1,2}^{\left(n\right)}\right)$ (see Supplemental Material [31]). The method of quaternions was used to resolve singularities at *θ*_2_ ± *π/*2 [32].

## Experiments

**Fig. 2:**
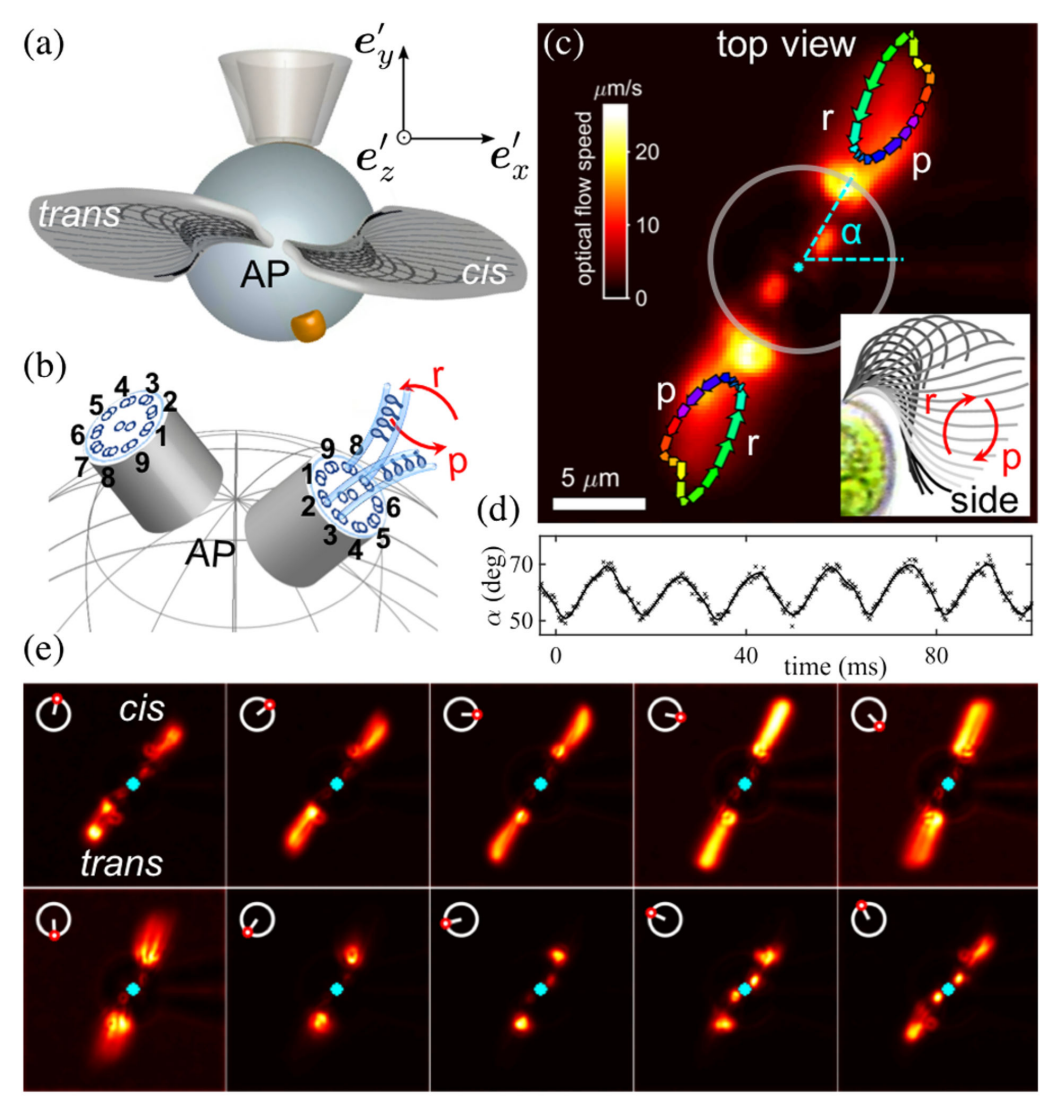
Nonplanar flagellar beating in *Chlamydomonas* (Supplemental Material, Video 1 [31]). (a) Cells aspirated onto pipettes are imaged from the anterior pole (AP). An eyespot is located ~45° from the mean beat plane. (b) The observed screwing motion of the flagella is coincident with the rotational symmetry of the basal apparatus, particularly, the chiral ordering of microtubule doublets 1 → 9. Known axonemal heterogeneities segregate the dyneins into two groups, those on doublets 2–4 operate for the power (*p*) stroke, but 6–8 for the recovery (*r*) stroke [40]. (c) Waveforms tracked by optic flow (arrows follow tip rotation) show a small offset [31] between the basal bodies. The instantaneous beat plane (defined by *α*) rotates periodically (d). Waveforms are ordered by phase [41] and averaged over ~1000 consecutive cycles to show the phase dependence of the nonplanar beat pattern (e).

Our model aims to link the three-dimensional nature of flagellar beating to the cell’s free-swimming behavior, but is *Chlamydomonas* flagellar beating truly nonplanar, and if so, to what extent? A possible out-of-plane component was detected based on manual tracings of flagellar waveforms [16]. The same authors argued that helical swimming could result from transient asynchronies between the two flagella. Yet this is incompatible with recent studies showing that phase slip asynchronies occur randomly, not periodically [26,33]. Here we use live-cell imaging and micromanipulation to prove that *Chlamydomonas* flagella beat with a persistent, nonplanar pattern *in vivo*. To better visualize the motion, we gently aspirated cells onto pipettes following previous protocols [33,39]. Cells were repositioned with flagella viewed (at 3000 frames*=*s) directly from above. The eye-spot delineates the *cis* (closest to the eyespot) from the *trans* flagella [Fig. 2(a)].

The results reveal that the waveform is inherently non-planar. The *Chlamydomonas* breaststroke is usually imaged in the $e_{x}^{\prime }-e_{z}^{\prime }$ plane [Fig. 2(c), inset]; here we observed a significant component of motion in the $e_{x}^{\prime }-e_{y}^{\prime }$ plane [Figs. 2(c)–2(e)]. During the power stroke, the flagella extend and pull away from the body while flagellar tips move in opposite directions; the direction of travel is immediately reversed during each subsequent recovery stroke. Over a stereotypical beat, the tips trace closed orbits [Fig. 2(c)]. The rotary motion generates axial torques, balanced by the micropipette. A freely swimming cell, viewed from above, will rotate clockwise (CW) during the power stroke but counterclockwise (CCW) during the recovery stroke. This is analogous to the “two steps forward, one step backward” interpretation for the in-plane breaststroke, which arises due to flagella drag anisotropy. We found that the rotary motion is also periodic (at the flagellar beat frequency) and is stable over thousands of cycles. We estimated the effective orbital tilt angle *β* from the high-speed videos using trigonometry: *β* = sin^−1^ [(𝓁*/ R*)] tan [Δ*α*/2]), where Δ*α* is the angle subtended by the flagellum’s uppermost tip in the transverse $e_{x}^{\prime }-e_{y}^{\prime }$ plane [31]. For *N* = 6 distinct cells, ~10^3^ consecutive beat cycles per individual, we estimated *β* = 0.3 rad ≈ 17.2° (see summary statistics in Table 1 in the Supplemental Material [31]).

## Simulations

We input this estimate of *β* into our three-bead model with cell-realistic parameters to determine if beat nonplanarity can indeed generate axial rotation and helical swimming. All lengths are nondimensionalized by 𝓁 (= 10 *μ*m, typical cell size), forces by the average tangential flagellar force *F*_0_ (= 30 pN, typical force produced by a flagellum [34]), and *η* by 10^−3^ pN *μ*m^−2^ (viscosity of water). When *β* = 0 (no tilt), pitch and roll (*θ*_1,3_) are suppressed, and our model reduces to the purely planar case investigated by other authors [24,42]. This is when the only nonvanishing component of torque is along $e_{y}^{\prime }$ [Eq. (4)] and swimming is constrained to the cell’s frontal plane (Ω_*y*_, yaw). For a nonplanar beat (*β* ≠ 0), there is a nonvanishing resultant axial torque *T*_*z*_. Equal driving forces $\left(F_{1}^{\left(t\right)}=F_{2}^{\left(t\right)}\right)$ produce purely axial rotation, since other components of **Ω** cancel by symmetry. The resulting trajectory is linear, with **Ω** ∥ ***V***. The trajectory can be a nondegenerate helix only if **Ω** ∦ ***V***. Rotational symmetry about $e_{z}^{\prime }$ can be broken if (1) the scaffold shape is asymmetric, (2) flagella planes have different tilt (*β*_1_ ≠ *β*_2_), and (3) flagella experience different driving forces $\left(F_{1}^{\left(t\right)}\neq F_{2}^{\left(t\right)}\right)$. The first two cases can produce irregular and nonhelical trajectories. We focus here on case 2 and only consider asymmetric force profiles of the form $F_{i}^{\left(t\right)}=1+c_{i}\cos\varphi_{i}$ [30,43].

For *β* > 0, symmetry breaking introduced by hydro-dynamic interactions between the beads results in either forward- or backward-CCW swimming at different phases of the beat cycle. The cycle-averaged swimming speed is given by $\bar{V}=\left(1/T_{b}\right){\int \;}_{0}^{T_{b}}V(t)dt=\left(1/T_{b}\right){\;\int \;}_{0}^{2\pi }[V(\bar{\varphi })/\dot{\bar{\varphi }}]/d\bar{\varphi }$ with flagellar period *T*_*b*_ and $\bar{\varphi }=\left(\varphi_{1}+\varphi_{2}\right)/2$, with an analogous expression for angular velocity $\bar{\Omega }$ [31]. The dynamical system described by Eqs. (2) and (3) is highly sensitive to swimmer shape. The strength of the hydrodynamic interactions between flagella beads are dictated by scaffold parameters, which determine net swimming (and rotation) direction per cycle, as in the 2D case [25,43]. We choose configurations where ${\bar{V}}_{z}>0,{\bar{\Omega }}_{z}<0$ (CCW is positive), and average flagella beat frequencies *f*_*b*_ and axial rotation ${\bar{\Omega }}_{z}$ fall within experimental values. See Supplemental Material [31] for further exploration of parameter space.

**Fig. 3:**
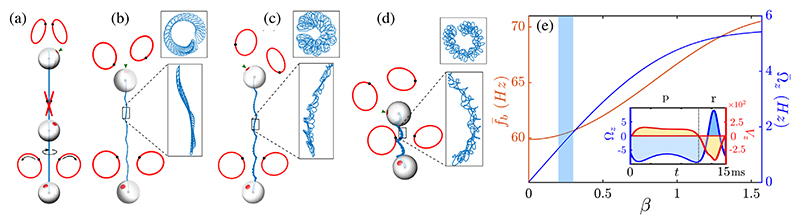
Nonplanar flagellar beating leads to superhelical trajectories (eyespot, small arrowhead). For the same geometry *a*_0_ = 0.53, *h* = 1.30, *R* = 0.60, *β* = 0.30, *a*= 0.05, examples show (a) purely axial rotation when *c*_1;2_ = 0.7, (b) “smooth” superhelix for *c*_1_ = 0.7, *c*_2_ = 0.71, (c) a superhelix with irregular inner helix for asymmetrical driving forces *c*_1_ = 0.6, *c*_2_ = 0.62, and (d) a more irregular trajectory for *c*_1_ = 0.3, *c*_2_ = 0.5. (e) Mean beat frequency of the two flagella (orange) and axial component of angular velocity (blue) as functions of *β* (numerical data fitted with sum of sines). The shaded region marks range of *β* measured in experiments (see Supplemental Material [31]). Inset: linear and angular velocities (in *μ*m*/*s and hertz) over one beat, note the CW-forward motion during the power (*p*) stoke and CCW-backward motion during the recovery (*r*) stroke.

We simulate free-swimming trajectories (Fig. 3) for a fixed scaffold shape (*a*_0_ = 0.53, *h* = 1.30, *R* = 0.60, *a* = 0.05), tilt angle *β* = 0.3, but different values of force asymmetry Δ*F* = *c*_2_ − *c*_1_. Figure 3(a) shows purely axial rotation (Δ*F* = 0). When Δ*F* ≠ 0, trajectories are *superhelical*. Superhelices emerge as general solutions of all low-Re swimming dynamics driven by periodic deformations [11] and have been observed in sperm swimming and in biaxial self-propelled particles under external torques [44]. A 1% difference in Δ*F* suffices to generate realistic superhelical trajectories [Fig. 3(b)]. Here, the average trajectory of the centroid ***r***_0_ prescribes an outer helix with a pitch of ~95 and radius ~5.9 *μ*m, while “fast” helical swirls appear on the timescale of the flagellar period $T_{b}=f_{b}^{-1}\approx 17\;\text{ms}$. Less regular helices emerge with higher asymmetry [Figs. 3(c) and 3(d)]. Frequencies of mean flagellar bead rotation *f*_*b*_ and axial rotation Ω_*z*_ vary with increasing orbital tilt angle *β* ∈ (0, 2*π*) [Fig. 3(e)]. As expected, axial rotation velocity increases with beat nonplanarity (higher *β*). A tilt angle *β* ≈ 0.3 corresponds to a rotation frequency of 1–2 Hz. We conclude that realistic helical swimming with a symmetric scaffold shape can be achieved using a very small force asymmetry between flagella, together with a small orbital tilt.

## Phototactic steering

**Fig. 4:**
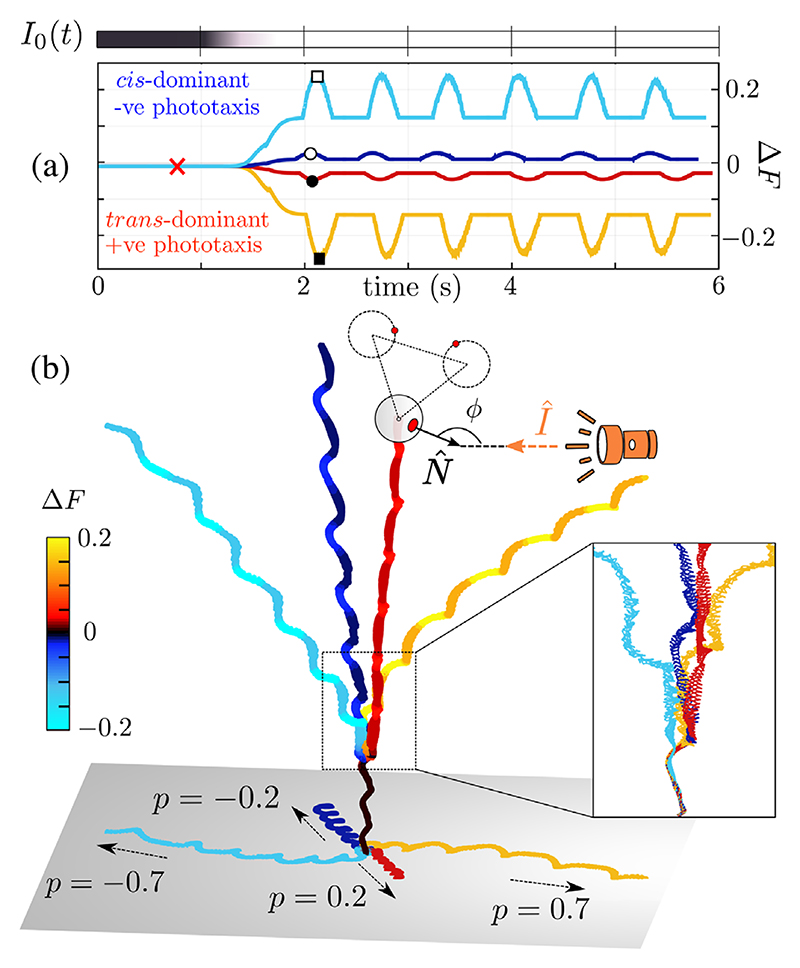
Flagellar dominance controls phototactic steering. (a) Time series of stimulus intensity *I*_0_ (gray scale = intensity) and the response Δ*F* for four different *p* values. Markers indicate characteristic *trans-cis* frequencies: times, 61–63; square, 58–75; filled square, 78–51; circle, 51–73; filled circle, 53–69 Hz. (b) The corresponding 3D tracks show either positive or negative phototaxis. Color indicates force asymmetry Δ*F*. In all cases, *a*_0_ = 0.53, *h* = 1.3, 𝓁 = 1, *R* = 0.6, *β* = 0.3, *a* = 0.05, *c*_cis_(*t* = 0) = 0.71, *c*_trans_(0) = 0.7.

Can cells exploit asymmetric flagellar driving for trajectory control? We hypothesize that this mechanism underlies phototaxis. *Chlamydomonas* can perform positive or negative phototaxis, depending on the stimulus [19,45]. Phototactic steering is associated with changes in both beat amplitude and frequency and is fine-tuned to the body rotation frequency [16,20,46]. Steering is accomplished by changing **Ω** [10,47], which in turn changes helix properties (radius, pitch, orientation). Here, **Ω** and *f*_*b*_ are strongly coupled. To simulate phototaxis (Fig. 4), we assume that a light stimulus **I** either attenuates or accentuates Δ*F* depending on the alignment between the eyespot and stimulus [48]. Denoting by $\hat{N}$ the vector normal to the eyespot surface, the intensity incident on the eyespot is $I(t)=I_{0}(t)\cos\phi (t)H[\cos\phi (t)]$, where $\cos\phi =\hat{N}\cdot \hat{I}=\;\left(e_{x}^{\prime }-e_{y}^{\prime }\right)\cdot \left(-e_{y}\right)/\sqrt{2}$, and *H* is the Heaviside step function. This framework can be applied to any type of taxis in response to a vectorial cue.

Flagella identity is important. When Δ*F* = 0.01 (no signal), the stronger (dominant) flagellum is the *cis* flagellum $\left(c_{\text{cis}}^{\left(0\right)}=0.71,c_{\text{trans}}^{\left(0\right)}=0.7\right)$. Force profiles are then modified to $c_{\text{cis,trans }}=c_{\text{cis,trans }}^{\left(0\right)}\mp p\log[1+I(t)]$, where *p* is the sensitivity of biflagellar dominance to the signal. Signs were chosen so that the model flagella responded differentially to the same signal [18] and in a direction compatible with experiments [49]. Importantly, here **Ω** emerges from the biflagellar force profiles, and was not assumed *a priori* to produce a regular helix. The resulting dynamics are characterized by a change in the pitch, radius, and axis of the (super)helical trajectory. The sign of flagellar dominance determines the sign of phototaxis. Compared to the no-light scenario, the cell turns toward the light when Δ*F* flips sign (*trans* dominant), but away when the *cis*-flagellum becomes more dominant (Δ*F* more positive). This indicates that alignment to a vectorial stimulus is indeed possible simply by varying the two relative forcing profiles (Fig. 4).

## Concluding remarks

We showed for the first time that *Chlamydomonas* flagella beat with a persistent, 3D beat pattern *in vivo* and quantified this as an orbital tilt. Accounting for this nonplanarity, we developed a three-bead hydrodynamic model with cell-realistic geometries, which captures the sense and magnitude of axial rotation of real cells. Superhelical trajectories emerged directly from the flagellar motion and were not prescribed. The model swims more slowly (15–30 *μ*m*=*s) than live cells (50–100 *μ*m*=*s [50]), suggesting limitations of a Stokeslet-type swimmer. To account for amplitude-frequency coupling pertaining to real flagella, future work will include elasticity, slender-filament dynamics, and basal coupling [39]. This can help constrain biflagellar synchrony to enhance swimming and steering efficiency.

In *Chlamydomonas*, the orbital tilt is likely to be fixed genetically. The beat pattern may result from an intrinsically chiral axoneme, which contains heterogeneities that could enforce bend anisotropy [40]. Such an axoneme may not be capable of sustaining perfectly planar beat patterns [11,51]. This view is supported by a recent study [52], which revealed that isolated *Chlamydomonas* axonemes, when reactivated with adenosine triphosphate (ATP), also beat with a three-dimensional pattern. Moreover, nearly all axonemes displayed the same torsional signature, consistent with the rotational symmetry of the basal apparatus and sense of flagellar rotation we observed [Figs. 2(b) and 2(c)].

A small asymmetry (1%) in biflagellar driving forces was necessary and sufficient for helical (not just purely axial) swimming. Whether this is augmented by further structural or shape asymmetries (if *β*_1_ ≠ *β*_2_) can be revealed by multifocal imaging, to measure the full torsional profile of each flagellum. A small *cis*-*trans* waveform asymmetry was already identified in the beat plane [33]. A stimulus-dependent asymmetry allowed our simple three-bead swimmer to perform phototaxis. Controllable steering is thus fairly insensitive to gait and the precise beating waveform. Indeed some algal biflagellates that do not use in-phase breaststrokes are nonetheless phototactic (e.g., *Polytoma, Cryptomonas*). Our Letter emphasizes the functional distinction between the two *Chlamydomonas* flagella, showing that a tunable and reversible biflagellar dominance likely operates in live cells for helical klinotaxis. Consistent with this view, mutant strains (*mia, bop*5-3, *ptx1*, and *lsp* 1) have “smoother” trajectories and defective or weakened photo-taxis [53–55]. Either flagellum can be dominant, depending on the stimulus. Indeed changes in Ca^2+^ produced opposite responses in reactivated *cis* and *trans* axonemes [18,49]. Our results offer key insights into the physiological basis of dynamic sensorimotor control, as implemented in a simple, aneural organism with few morphological asymmetries. An important next step will be to determine how biflagellar dominance is regulated at the molecular level (e.g., of dynein activity).

We thank Ray Goldstein for support during the initial phase of this project and Peter Ashwin and Gáspár Jékely for useful discussions. This work was funded by the European Research Council (ERC) under the European Union’s Horizon 2020 Research and Innovation Programme (Grant No. 853560, *EvoMotion*) and a Springboard Award from the Academy of Medical Sciences and Global Challenges Research Fund (SBF003\1160) to K. Y. W.

## Supplementary Material

Supplementary Material
